# A Machine-Learning Model for Lung Age Forecasting by Analyzing Exhalations

**DOI:** 10.3390/s22031106

**Published:** 2022-02-01

**Authors:** Marc Pifarré, Alberto Tena, Francisco Clarià, Francesc Solsona, Jordi Vilaplana, Arnau Benavides, Lluis Mas, Francesc Abella

**Affiliations:** 1 Department of Computer Science & INSPIRES, University of Lleida, Jaume II 69, 25001 Lleida, Spain; mpm4@alumnes.udl.cat (M.P.); francisco.claria@udl.cat (F.C.); jordi@diei.udl.cat (J.V.); arnaubenavides97@gmail.com (A.B.); lluis.mas@udl.cat (L.M.); 2 CIMNE, Building C1, North Campus, UPC, Gran Capità, 08034 Barcelona, Spain; atena@cimne.upc.edu; 3 IRBLleida, Avda Alcalde Rovira Roure 80, 25198 Lleida, Spain; abellapons@gmail.com

**Keywords:** exhalation, lung capacity forecasting, machine learning

## Abstract

Spirometers are important devices for following up patients with respiratory diseases. These are mainly located only at hospitals, with all the disadvantages that this can entail. This limits their use and consequently, the supervision of patients. Research efforts focus on providing digital alternatives to spirometers. Although less accurate, the authors claim they are cheaper and usable by many more people worldwide at any given time and place. In order to further popularize the use of spirometers even more, we are interested in also providing user-friendly lung-capacity metrics instead of the traditional-spirometry ones. The main objective, which is also the main contribution of this research, is to obtain a person’s lung age by analyzing the properties of their exhalation by means of a machine-learning method. To perform this study, 188 samples of blowing sounds were used. These were taken from 91 males (48.4%) and 97 females (51.6%) aged between 17 and 67. A total of 42 spirometer and frequency-like features, including gender, were used. Traditional machine-learning algorithms used in voice recognition applied to the most significant features were used. We found that the best classification algorithm was the Quadratic Linear Discriminant algorithm when no distinction was made between gender. By splitting the corpus into age groups of 5 consecutive years, accuracy, sensitivity and specificity of, respectively, 94.69%, 94.45% and 99.45% were found. Features in the audio of users’ expiration that allowed them to be classified by their corresponding lung age group of 5 years were successfully detected. Our methodology can become a reliable tool for use with mobile devices to detect lung abnormalities or diseases.

## 1. Introduction

Respiratory diseases cause immense health, economic and social costs and are the third cause of death worldwide [1] and a significant burden for public health systems [2]. Significant research efforts have been dedicated to improving early diagnosis and monitoring of patients with respiratory diseases to allow for timely interventions [3]. Respiratory sounds are important indicators of respiratory health and disorders [4]. Distinction between normal respiratory sounds and adventitious ones (such as crackles, wheezes or squawks) is important for an accurate medical diagnosis [5,6].

Spirometry is generally performed in care centres using conventional spirometers, but home spirometry with portable devices is slowly gaining acceptance [7]. Home spirometry has the potential to result for earlier treatment of exacerbations, more rapid recovery, reduced health care costs, and improved outcomes [8]. Challenges currently faced by home spirometry are cost, patient compliance and usability, and an integrated method for uploading results to physicians [9]. Digital techniques applied to home spirometry using personal computers or mobile devices (i.e. smartphones), often increase overall performance.

Spirometry is the most widely employed objective measure of lung function [10]. A standard spirometer measures flow the rate of air as it passes through a mouthpiece. The four most common clinically-reported measures are FVC (Forced Vital Capacity), FEV1 (Forced Expiratory Volume in one second), FEV1/FVC, and PEF (Peak Expiratory Flow), as they are used to quantify the degree of airflow limitation in chronic lung diseases such as asthma, COPD, and cystic fibrosis. A healthy individual’s lung function measurements, taken with spirometry, are generally at least 80% of the values predicted based on age, height and gender [11]. In addition, the frequency pattern of the spectrogram can be obtained. A speech signal contains periodic components with fundamental frequencies ranging between 85 and 255 Hz [12].

Many hardware devices, coupled to smartphones have been designed [13]. However, we are interested on designing a spirometer app for a smartphone, without additional hardware, gadgets or whistles. Larson et al. [14] first showed that it is possible to perform a spirometry test using a smartphone. Jointly with Spirocall [15], they extracted separate feature sets for FEV1, FVC, and PEF from these time-domain flow-rate estimations. For example, for a given flow-rate estimate, they obtained the maximum value and used it as a feature for PEF regression. Integrating the flow-rate estimate with respect to time, a feature for FVC regression was obtained. Using this approach, 3 sets of 38 features for FEV1, FVC, and PEF, each, were generated. The mean error for these three metrics when both used specific whistles was 5.1% on average in Spirosmart and 8.3% in Spirocall for these three metrics. The sounds of the spirometry-efforts were delivered across Internet in Spirosmart. In Spirocall instead, a standard telephony voice channel (GSM) was used to transmit the efforts. It was argued that individuals in low- or middle-income countries do not typically have access to the latest smartphones. The authors in [16], found that the relation between the mean of frequency responses was in the range of 100 HZ to 1200 HZ and the flow rate had the highest correlation factor of 0.8913 among other possible relations. Regression analysis was performed on the collected data and the quadratic regression technique gave the lowest Root Mean Square (RMSE) among other possible regressions.

However, smartphone spirometry is particularly susceptible to poorly performed efforts because any environmental noise (e.g., a person’s voice) or mistakes in the effort (e.g., coughs or short breaths) can invalidate the results. The authors in [17] used two Neural Network models fed by Mel-spectrogram features to analyze and estimate the quality of smartphone spirometry efforts. A gradient boosting model achieved 98.2% accuracy at identifying invalid efforts when given expert tuned audio features, while a Gated-Convolutional Recurrent Neural Network reached an accuracy of 98.3%.

When performing a spirometry, the lung age value was introduced in 1985 and Kristen Deane in [18] stated that, the lung age is the average age of a non-smoker with an FEV1 equal to theirs, concluding that quit rates are higher when patients know their lung age. For instance, at 1 year, verified quit rates were 13.6% in the intervention group and 6.4% in the control group (a difference of 7.2%, 95% CI; *p* = 0.005). This means that for every 14 smokers who are told their lung age and shown it on a graphic display, almost one additional smoker will quit after 1 year.

We present an environment for diagnosing lung malfunction by obtaining lung-age, instead of the FEV1, FVC, and PEF measures, as in [14,15]. The main reason for this is the need to avoid noise. Users should be careful to take samples in completely noise-free places or to apply mechanisms to know the goodness (freedom from noise) of the spirometry sample. In addition, lung age is easier to understand and more convincing than traditional metrics to set off an individual’s alarm when suffering from a lung problem. This would be reflected in the fact that lung and real age are quite far apart.

Our main objective is to find time-frequency features and preprocess a data corpus to feed a machine-learning model to predict the lung age. Our main contribution is the group of features found with which the maximum performance was obtained. Apart from age, two groups of features are studied. One is the called spirometer-like features, trying to emulate the FVC, FEV1 and PEF metrics used in spirometry. We inspired on the studies performed in [14,15] to estimate the lung age. The other group, called time-frequency features, try to capture frequency patterns by taking into account the spectrogram of a cold blowing signal [19], the kind of blowing made in an exhalation. This set of features also take into account different frequency bands, and thus, the range of frequencies where the cold blow is located, could increase even more its significance.

Main expectation is to obtain the lung age of a user through the extraction of the features from an exhalation record. We propose a collection of features from lightweight extraction of an exhalation for determining lung age. High social and clinical benefits in obtaining an approximation to lung age with a simple, easy and cheap process by implementing it in current and widely spread smartphones, would give the opportunity for the method to be used worldwide by people of any income level.

## 2. Methods

### 2.1. Corpus

One of the most important phases of the methodology is the corpus sampling. The accuracy of the modelled classifiers depends enormously on the length and annotation of the corpus.

**Figure 1:**
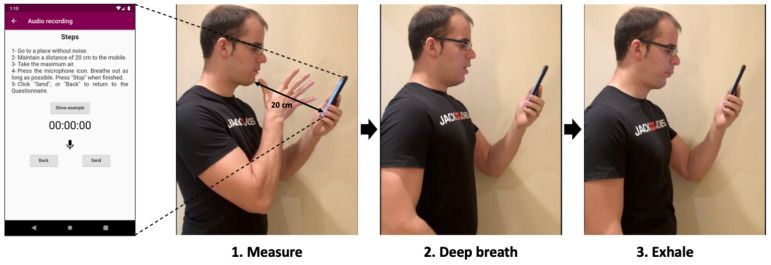
Data collection process. The participant opens the app, moves the phone to the indicated distance (20 cm), takes a deep breath and exhales.

A mobile app and an analogous website for registering exhalations was implemented. Sampling records (Figure 1) consists of maintaining a distance of approximately 20 cm between the mouth and the phone. Then, the user takes a deep breath and exhales with as much force as possible for as long as possible. In addition, a support video sample was provided. This is in line with traditional spirometry.

There were a total of 188 user samples. For each sample, 42 features were obtained, classified into three different types. The first type includes a basic demographic feature, gender. The samples were almost evenly split by gender. There were 91 men (48.4%) and 97 women (51.6%). The age range of the participants was between 17 and 67 years old, with an average age of 40.8 years for men and 44.9 for women. The remaining feature types were the so-called spirometer-like and time-frequency features.

### 2.2. Features

**Gender** was considered as an important feature to be taken into account. In addition, two more groups of features were used. One group consists of **spirometer-like** features related to the measures taken in a traditional spirometry by analyzing volume-flow representation. The second group contains additional **time-frequency** features, to find possible patterns or specific marks of the time-frequency representation of the exhalations.

#### 2.2.1. Spimoreter-Like Features

**Figure 2:**
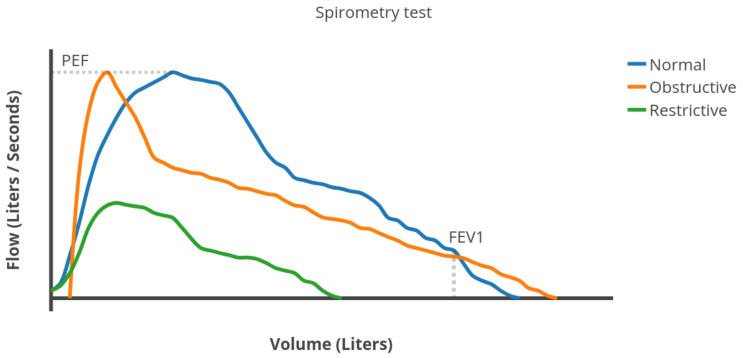
Example of different flows rates in a spirometry test.

During a spirometry test, the patient takes the deepest breath possible and then exhales with as much force as possible for as long as possible. The spirometer calculates various lung function measures based on the test [15]. Three of the most important ones are (see Figure 2):

1. Forced Vital Capacity (FVC): the total volume of air expelled during the expiration.
2. Forced Expiratory Volume in one second (FEV1): the volume of air expelled in the first second of expiration.
3. Peak Expiratory Flow (PEF): the maximum expiratory flow rate reached during the exhalation.

Figure 2 shows an example of flow vs. volume plot, generated by a spirometer [11,20]. FVC plot is similar to an exponential density function (blue line in Figure 2) in healthy people. As obstruction the airflow increases, the flow rate decreases faster than exponentially after reaching its maximum value (PEF). Therefore, the distribution becomes as the orange line in Figure 2. When suffering from a restrictive lung disease, the respiratory muscles weaken and the lung capacity (FVC) decreases (green line in Figure 2). The shape is very similar to a Weibull distribution.

In order to approximate FEV1, FVC and PEF, three easily measurable features in mobile devices were used instead. These features were obtained using a python library named *Librosa* [21], used to process audio. Using this library, the Short-time Fourier transform (STFT) was applied to each audio to decompose the audio wave into a time-frequency spectrogram. Then, a transformation to convert the wave amplitude values of the spectrogram into decibels was used to obtaining the following features:

- **Total_dec**: The summation of all the decibels of the audio over all frequencies as an absolute value. This is an approximation to FVC.
- **Total_dec_1st_sec**. The sum of all the decibels during the first second of the audio over all frequencies as an absolute value. This is an approximation to FEV1.
- **Max_peak**. The maximum peak of decibels from all the audio and frequencies. This is an approximation to PEF.

#### 2.2.2. Time-Frequency Features

When a person blows, the vocal cords are inactive. Only the position of the mouth affects the nature of the emitted sound [19]. The emission of a blowing sound can thus be approximated by a white noise source passing through a band-pass filter. Two different blowing sound types are considered, labeled as hot and cold blowing sounds. A hot blowing sound is the type made by someone trying to mist up a window. A cold blowing sound is, for example, the sound of someone cooling off a bowl of soup. The hot blowing exhalation sounds are comprehended in frequency bands between 450 Hz and 1300 Hz and the cold blowing between 1500 Hz and 4000 Hz [19].

The energy for both blowing sound types is clearly located in different parts of the spectrum. Normal spirometries are expected to have a cold-frequency pattern [12]. Thus, we focused on defining time-frequencies able to distinguish this particularity of such cold sounds.

Due to the good results obtained in [22], the same features were used to find patterns and related marks to identity hot sounds from cold ones. Furthermore, it was shown that they captured the pattern of ALS patients with bulbar involvement very well. Thus, it seems reasonable that this set of features also captures frequency aspects of diseases affecting exhalation power. Furthermore, the frequencies in a cold blow are mainly concentrated in the 1500–4000 Hz range. This tells us that splitting features into different bands, as was proposed in [22], could be a good decision.

Firstly, the Wigner distribution (WD) of the real signal x(t) of each voice segment was obtained and convoluted with the Choi–Williams exponential function. The resulting Choi–Williams distribution was normalized ($CWD_{N}\left(f,t\right)$). For more details see [22].

Then, the joint probability density distribution pD(f,t) (Equation (1)) was obtained.

(1) $$pD\left(f,t\right)=m_{t}\left(t\right)\cdot m_{f}\left(f\right),$$

where $m_{t}\left(t\right)$ and $m_{f}\left(f\right)$ are the marginal density functions of $CWD_{N}\left(f,t\right)$.

A total of 38 time-frequency features were used. 28 were obtained over a wide range of 7 frequency bands (0–80 Hz, 80–250 Hz, 250–550 Hz, 550–900 Hz, 900–1500 Hz, 1500–3000 Hz and 3000–44,100 Hz). This separation was based on the range of frequencies of the cold blow. These are:

- **E_Bn1…E_Bn7**: average of the instantaneous spectral energy E(t) (Equation (2)) of each sample, for each 7-bands.
  (2) $$E\left(t\right)=\int_{f_{1}}^{f_{2}}pD\left(f,t\right)df,$$
  where $f_{1}$ and $f_{2}$ are the lower and upper frequencies of each band.
- **f_Cres1…f_Cres7**: average of the Instantaneous Frequency Peak, f_Cres(t) (Equation (3)), for each 7-bands.
  (3) $$f\_Cres\left(t\right)=\frac{1}{E(t)}argmax_{f}\left[\prod_{f_{1}}^{f_{2}}f\cdot pD\left(f,t\right)\right]$$
- **f_Med1…f_Med7**: average of the instantaneous frequency $f_{mi}\left(t\right)$ (Equation (4)), for each 7-bands.
  (4) $$f_{mi}\left(t\right)=\int_{f_{1}}^{f_{2}}\frac{1}{E(t)}fpD\left(f,t\right)df$$
- **IE_Bn1…IE_Bn7**: average of the spectral information, IE(f) (Equation (5)), for each 7-bands.
  (5) $$IE\left(f\right)=-log_{2}\left(m_{fN}\left(f\right)\right)$$

The remaining features were obtained using the entire frequency range of the audios (0–44,100 Hz):

- ***H_t***: instantaneous entropy (Equation (6)).
  (6) $$H\_t=-\int log_{2}\left(m_{tN}\left(t\right)\right)\cdot m_{tN}\left(t\right)dt$$
- ***H_f***: spectral entropy (Equation (7)).
  (7) $$H\_f=-\int log_{2}\left(m_{fN}\left(f\right)\right)\cdot m_{fN}\left(f\right)df$$
- ***H_tf***: joint Shannon entropy in a range of 0 to 20 bits (Equation (8)).
  (8) H_tf=H_t+H_f
- **K**: Kurtosis (Equation (9)).
  (9) $$K=\langle m_{t}{\left(t\right)}^{4}m_{f}{\left(f\right)}^{0}\rangle$$
- The joint time-frequency moments $\langle t^{n}f^{m}\rangle$ for n=1 and m=1 (**momC11**), n=7 and m=7 (**momC77**) and n=15 and m=15 (**momC15**).
- The same joint moments of the marginal signals of instantaneous power and spectral density $\langle m_{t}{\left(t\right)}^{n}m_{f}{\left(f\right)}^{m}\rangle$ (**momM11**, **momM77** and **momM15**) were also considered.

### 2.3. Prediction Models

Several machine-learning algorithms can be used to obtain good predictions. The most common algorithms in sound recognition were used. These were K-Nearest Neighbors (K-NN), C-Support Vector Classification (C-SVC), Random Forest (RF), Decision Tree (DT), Naïve Bayes (NB), Logistic Regression (LR), Linear Discriminant Analysis (LDA) and Quadratic Discriminant Analysis (QDA). In order to figure out which of these algorithms predicts better over our set of features, the scikit-learn machine-learning library [23] was used to implement and analyze these algorithms.

K-NN is a non-parametric classification method [24]. The output is classified by the most common one among its K nearest neighbors. While there are a number of different types of the popular Support Vector Machine (SVM) algorithms, for the purpose of this research, we used C-SVC, because it can incorporate different basic kernels [25]. C-SVC is thought to solve biomedical problems in a variety of clinical domains. RF consists of many decision trees that it use ensemble learning [26]. It was implemented with a forest of 300 decision tree predictors. DT is very similar to RF. It consists of a tree structure, where each internal node denotes a test on an attribute. Each leaf represents an outcome of the test [27]. NB is a probabilistic classifier based on applying Bayes’ theorem with strong (naïve) independence assumptions between the features [28]. LR is one of the algorithms most widely used for regression but it is also used for classification or predicting problems. It is based on a sigmoid function and works best on binary classification problems [29]. Despite its simplicity, LDA often produces robust, decent, and interpretable classification results. When addressing real-world classification problems, LDA is often the benchmarking method used before other more complicated and flexible ones [30]. QDA [31] is a variant of LDA in which an individual covariance matrix is estimated for every class of observation. QDA is particularly useful if there is prior knowledge that individual classes exhibit distinct covariances.

### 2.4. Performance Metrics

There are several metrics for evaluating classification algorithms [32]. The analysis of these metrics and their significance must be interpreted correctly to evaluate these algorithms.

There are four possible results in the classification task. If the sample is positive and is classified as such, it is counted as a *true positive* (*TP*) and when classified as negative, it is considered a *false negative* (*FN*). If the sample is negative and it is classified as negative or positive, it is considered a *true negative* (*TN*) or *false positive* (*FP*), respectively. Based on that, the three performance metrics presented below were used to evaluate the performance of the classification models.

- **Accuracy** (Equation (10)). The ratio between the correctly classified samples.
  (10) $$\mathrm{Accuracy}=\frac{TP+TN}{TP+TN+FP+FN}$$
- **Sensitivity** (Equation (11)). The proportion of correctly classified positive samples compared to the total number of positive samples.
  (11) $$\mathrm{Sensitivity}=\frac{TP}{TP+FN}$$
- **Specificity** (Equation (12)). The proportion of correctly classified negative samples compared to the total number of negative samples.
  (12) $$\mathrm{Specificity}=\frac{TN}{TN+FP}$$

Finally, paired Bonferroni-corrected Student *t*-tests [33] were implemented to evaluate the statistical significance of the metrics results. The null hypothesis consists of considering that there is no difference in the performance of the classifiers. The tests with *p*-values below 0.05 rejected the null hypothesis.

## 3. Results

### 3.1. Prescreening

The data corpus was previously curated in order to obtain performance outcomes that were as high as possible.

#### 3.1.1. Correlation Analysis

First of all, the correlation between all the features was found. This was implemented by using the Pandas library [34]. Next, the correlation matrix with the overall dataset among all the features was computed. The features that exceeded a correlation of 90% were discarded. In this case, only the H_f feature was discarded.

#### 3.1.2. Principal Component Analysis

The PCA (Principal Component Analysis) was applied next in order to reduce the number of features. The objective was to have the best Accuracy by using only the most significant features. This not only tended to raise performance results but in addition, to speed up the classification algorithm. Speeding up the classification process may be a mandatory requirement if, for practical reasons, the method must be implemented on a mobile device, with reduced computing power. To perform this PCA analysis, the scikit-learn [23] library for python was also used.

After applying PCA, the features that explained 99.9% of the corpus were chosen. The number of features was reduced from 41 to 29. These were gender, Total_dec, Max_peak, E_Bn (bands 2, 3, 5 and 6), IE_Bn (bands 2, 3, 5 and 7), f_Cres (bands 2, 3, 4, 5 and 6), f_Med (bands 1, 2, 4, 5, 6 and 7), H_t, H_tf, K, momC11, momC77, momM11 and momM77. The remaining features, which explained the other 0.1%, were discarded.

#### 3.1.3. Oversampling

**Figure 3:**
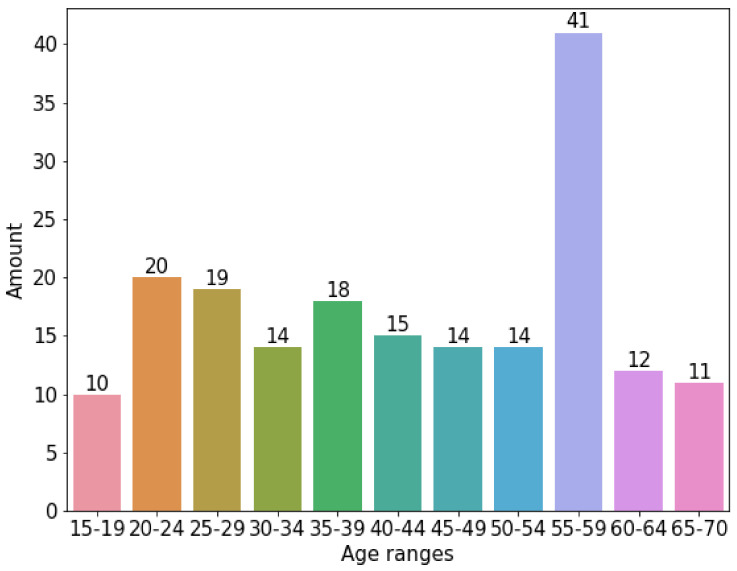
Original data corpus distribution among range-ages without using SMOTE.

Figure 3 shows the corpus used. As can be appreciated, it is clearly small and unbalanced. An oversampling algorithm was used. This was SMOTE [35] (Synthetic Minority Oversampling Technique), which consists of duplicating samples without adding new information, so that these new synthetic registers can be added to the corpus data. This algorithm is very efficient for adding as much synthetic data as required to balance the data corpus.

**Figure 4:**
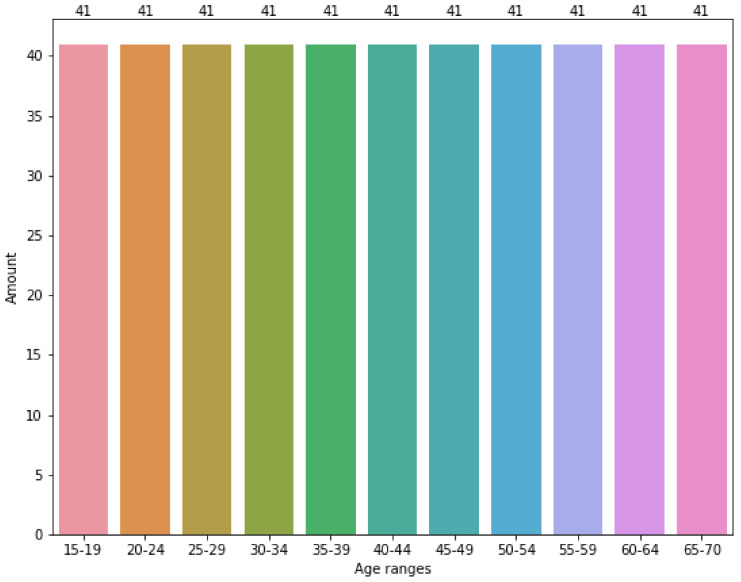
Original data corpus distribution among range-ages using SMOTE.

Figure 4 shows the effects of applying the SMOTE algorithm to the original data corpus split in groups of 5 years. As can be appreciated, the data are evenly distributed between the age groups. Basically, SMOTE introduced additional synthetic data to the corpus. For each age group, the synthetic data added is the difference between the number of registers in Figure 4 minus the number of registers in the same column of Figure 3.

### 3.2. Group Classification

**Figure 5:**
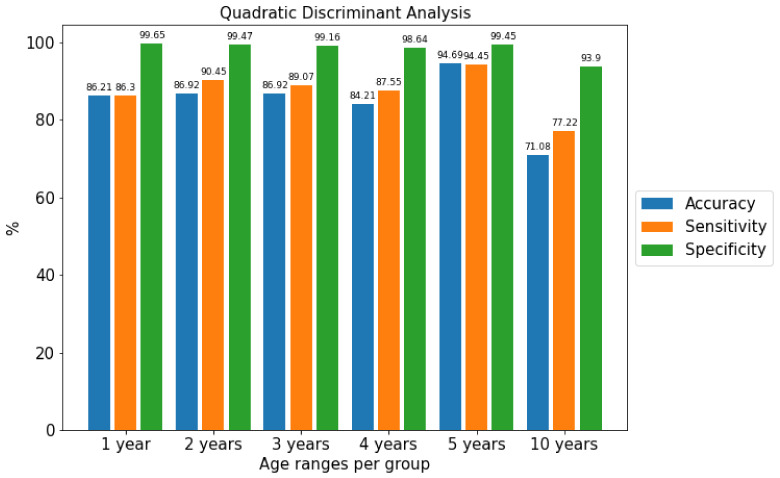
Comparison of the metrics of the best algorithm separated by age ranges using the Quadratic Discriminant Analysis algorithm.

Experiments were performed varying the number of age ranges from the youngest user, 17 years old, to the oldest, aged 67. The ranges chosen were 1, 2, 3, 4, 5 and 10 years. For the different classification algorithms and these groups of age from the corpus, the Accuracy, Sensitivity and Specificity performance metrics were obtained independently of gender. As can be seen in Figure 5, the age range grouped into sets of 5 years obtained the best results.

### 3.3. Classification Accuracy

After preparing the definitive corpus, a set of machine-learning algorithms were tested. The group range with the best metrics (5 years) was used with and without applying oversampling (i.e., the SMOTE algorithm). The outcomes shown in Table 1 were obtained. In general, the use of SMOTE significantly improved the Accuracy, Sensitivity and Specificity of the classification algorithms. The Quadratic Discrimination Analysis (QDA) was the machine-learning algorithm that performed best when SMOTE was applied. Note that the outcomes when SMOTE was not applied are very incoherent, irregular, with no sense. This shows that oversampling also improves the coherence of the result from the algorithms.

The results were also obtained for men and women separately by applying SMOTE (Table 2). In the men and women cases, it can be observed that QDA also obtained the best outcomes. With an Accuracy of 94.69%, Sensitivity of 94.45% and Specificity of 99.45%, we can see that it is better to treat the audios together rather than separately by gender. For example, when treating only men (Accuracy = 74.29%, Sensitivity = 74.67%, Specificity = 97.16%) or women (Accuracy = 91.67%, Sensitivity = 92.73%, Specificity = 99.16%)), the metrics dropped below the ones obtained jointly.

## 4. Discussion

The good results obtained demonstrate that it is possible to obtain the lung age of a user by extracting the features from an exhalation. The collection of lightweight features proposed were enough to supply particularities and patterns of exhalations. Most of them turned out to be very significant. Applying features successfully for the detection of bulbar ALS has also proven to be efficient in classifying lung age ranges according to exhalations.

The cold properties of exhalations were successfully captured by dividing them between different frequency bands. Balancing age groups with additional synthetic data increased overall performance notably. In general, the outcomes correlated with the size and improvement of the data corpus. No gains were observed when the model was applied separately to males or females.

The Accuracy, Sensitivity and Specificity we obtained in measuring lung age were 94.69%, 94.45% and 99.45%, respectively. With an error of 5.31% (Accuracy), the outcomes show that the method is suitable for implementation and deployment in websites or mobile devices. The latter option could require some additional computing support.

### 4.1. Comparison with Previous Work

The utility of treating patients by obtaining and subsequently displaying lung age has been demonstrated to be effective [18]. In addition, its use has also been validated [36].

Due to the non-existence of similar studies predicting lung age using a smartphone, we had to compare accuracy and other metrics with other studies that obtain the main features used in a spirometry (FEV1, FVC and PEF). As reflected in Section 2.2.1, we attempted to emulate these features. Thus, we consider it relevant to compare our results with similar research that used these features. The results obtained are similar to those reached in [14,15], where the average mean error, when specific whistles were used, was 5.1% in Spirosmart and 8.3% in Spirocall, for the three common spirometer-measures (FEV1, FVC and PEF).

Our target is to inform the user lung age, instead of the measures FEV1, FVC and PEF. This makes it easier for people who are not clinical experts to interpret the results. A low average error of 5.31% was obtained when using 5-year groups. An Accuracy of 94.69% and Sensitivity of 94.45% means that almost all correctly classified patients correspond to the correct group and the Specificity of 99.45% means that almost all decisions not to classify a patient at the wrong lung age are correct. As can be seen in Table 3, the results are very similar to those from the other applications mentioned above, with a notable difference in the number of samples. Thus, we can conclude that our study is reliable enough to be used.

### 4.2. Limitations

It was not possible to have a fully balanced dataset, thus oversampling techniques had to be used to improve the results. This means that with a larger and more balanced dataset, the results could have been improved and a more accurate lung age would have been predicted. A small corpus could also be the cause of the poor results obtained when dealing with men and women separately. More research with a more curated and enlarged dataset should be done to tackle this issue.

## 5. Conclusions

This article presents a methodology for determining the lung age of a person blowing on the microphone of any kind of recording device (i.e., smartphone). It is designed to allow self-lung controls or help clinicians to follow-up patients in order to avoid unnecessary hospital visits as well as health resources.

The results are better than expected as we have been able to emulate the behavior of a spirometer with results in line with the literature, which always shows an accuracy of over 90% and an error rate between 5% and 8%. Although much work has been done to emulate a spirometry, we can say that we have made a first satisfactory approximation to predicting lung age. This will increase self-control of the lung function because the results provided are more popular than traditional spirometry metrics. This, jointly with the widespread use of smartphones worldwide, can increase the early detection and treatment of lung diseases.

As a future trend, the aim is to improve our current dataset of audio samples to improve the results and reliability and further narrow the age range.

## Figures and Tables

**Table 1:** Machine-learning Accuracy (Acc.). Sensitivity (Sen.) and Specificity (Spe.) with and without SMOTE.

Classifiers	No SMOTE			SMOTE		
	Acc.	Sen.	Spe.	Acc.	Sen.	Spe.
K-NN	4.26%	10.91%	90.07%	50.44%	56.26%	95.50%
C-SVC	25.53%	9.09%	90.91%	4.43%	9.09%	90.91%
DT	17.02%	19.78%	91.36%	60.18%	64.75%	95.99%
RF	12.77%	6.15%	90.57%	74.34%	77.89%	97.45%
NB	10.64%	16.00%	91.07%	42.48%	47.11%	94.24%
LR	14.89%	6.91%	90.64%	38.05%	43.87%	93.91%
LDA	6.38%	3.90%	90.33%	50.44%	53.86%	95.05%
QDA	12.77%	8.20%	90.97%	**94.69**%	**94.45**%	**99.45**%

**Table 2:** Machine-learning Accuracy (Acc.), Sensitivity (Sen.) and Specificity (Spe.) with SMOTE by gender.

Classifiers	Men			Women		
	Acc.	Sen.	Spe.	Acc.	Sen.	Spe.
K-NN	60.00%	59.00%	95.48%	66.67%	67.05%	96.63%
C-SVC	20.00%	35.00%	91.52%	4.17%	9.09%	90.91%
DT	45.71%	48.00%	93.85%	65.28%	64.77%	96.57%
RF	62.86%	63.50%	95.84%	77.78%	79.48%	97.80%
NB	57.14%	59.00%	95.21%	54.17%	57.88%	95.37%
LR	48.57%	49.50%	94.24%	47.22%	53.33%	94.77%
LDA	57.14%	57.00%	95.22%	66.67%	68.34%	96.67%
QDA	**74.29**%	**74.67**%	**97.16**%	**91.67**%	**92.73**%	**99.16**%

**Table 3:** Comparison with other applications in the literature [14,15].

	Error Rate	Accuracy	Samples
SpiroSmart	5.10%	94.90%	50
SpiroCall	8.30%	91.70%	53
Spirometer (lung-age)	5.31%	94.69%	188

## Data Availability

Data Repository with the features used to obtain the results exposed in the manuscript can be found in a GitHub repository in https://github.com/mpifa/Spirometer, accessed on 29 December 2021.

## Sample Availability

Source and Synthetic Data Repository. All the models implemented in python are available in a GitHub repository in *Spirometer model and dataset* (https://github.com/mpifa/Spirometer, accessed on 29 December 2021). A dataset of the features obtained is also provided. It was built in python from the original values.

## Abbreviations

The following abbreviations are used in this manuscript:

LDA	Linear Discrimination Analysis.
QDA	Quadratic Linear Analysis.
PCA	Principal Component Analysis
SMOTE	Synthetic Minority Oversampling Technique.
FVC	Forced Vital Capacity
FEV1	Forced Expiratory Volume in one second
PEF	Peak Expiratory Flow
COPD	Chronic obstructive pulmonary disease
GSM	Global System for Mobile communications
RMSE	Root Mean Square
CEIM	Research Ethics Committee for Biomedical Research Projects
ALS	Amyotrophic lateral sclerosis
STFT	Short-time Fourier transform
WD	Wigner distribution
CWD	Choi–Williams distribution
K-NN	K-Nearest Neighbor
C-SVC	C-Support Vector Classification
RF	Random Forest
DT	Decision Tree
NB	Naïve Bayes
LR	Logistic Regression
LDA	Linear Discriminant Analysis
QDA	Quadratic Discriminant Analysis
TP	True Positive
FN	False Negative
FP	False Positive
TN	True negative
